# Academic contributions to the development of evidence and policy systems: an EPPI Centre collective autoethnography

**DOI:** 10.1186/s12961-023-01051-0

**Published:** 2023-10-25

**Authors:** Sandy Oliver, Kelly Dickson, Mukdarut Bangpan

**Affiliations:** Social Research Institute, University College London, 10 Woburn Square, London, WC1H 0NR United Kingdom

**Keywords:** Evidence systems, Policy development, Collaboration, Co-production, Autoethnography

## Abstract

**Background:**

Evidence for policy systems emerging around the world combine the fields of research synthesis, evidence-informed policy and public engagement with research. We conducted this retrospective collective autoethnography to understand the role of academics in developing such systems.

**Methods:**

We constructed a timeline of EPPI Centre work and associated events since 1990. We employed: Transition Theory to reveal emerging and influential innovations; and Transformative Social Innovation theory to track their increasing depth, reach and embeddedness in research and policy organisations.

**Findings:**

The EPPI Centre, alongside other small research units, collaborated with national and international organisations at the research-policy interface to incubate, spread and embed new ways of working with evidence and policy. Sustainable change arising from research-policy interactions was less about uptake and embedding of innovations, but more about co-developing and tailoring innovations with organisations to suit their missions and structures for creating new knowledge or using knowledge for decisions. Both spreading and embedding innovation relied on mutual learning that both accommodated and challenged established assumptions and values of collaborating organisations as they adapted to closer ways of working. The incubation, spread and embedding of innovations have been iterative, with new ways of working inspiring further innovation as they spread and embedded. Institutionalising evidence for policy required change in both institutions generating evidence and institutions developing policy.

**Conclusions:**

Key mechanisms for academic contributions to advancing evidence for policy were: contract research focusing attention at the research-policy interface; a willingness to work in unfamiliar fields; inclusive ways of working to move from conflict to consensus; and incentives and opportunities for reflection and consolidating learning.

## Background

Research is increasingly informing policy, practice and personal decisions, encouraged by three intersecting fields: research synthesis; evidence-informed decision-making; and public engagement with research. The research synthesis field started in 1970s with an explosion of new statistical methods for pooling data from multiple studies [100] to increase confidence in research findings. Over successive decades, methods diversified across academic disciplines, particularly in social science where syntheses addressed different types of questions and literatures [101]. Thus, various forms of evidence became available for the second field, evidence-informed decision-making, dubbed evidence-based medicine in Canada [57] before entering education in the UK [16] and then other sectors, leading to the development and embedding of decision-makers’ skills and procedures to engage with research and researchers [46]. As evidence increasingly influenced decision-making, it attracted interest from the third field, public engagement with research, which has long histories in the health sector [11] and in higher education, science and technology, public policy, development studies and community development [28]. Thus research evidence became more relevant to public perceptions of problems and policy options. These three fields, of research synthesis, evidence-informed decision-making and public engagement with research, have advanced different aspects of the evidence movement often by people working across the conventional boundaries imposed by academic disciplines and policy sectors.

We, the authors of this autoethnography, have worked across all three fields, advancing methods for synthesising research, collaborating with teams making decisions for policy, professional practice and personal lives and, since 2007, strengthening capacity in systematic reviewing for policy priorities in the global south. These fields all feature in the literature about how evidence has become an integral and sustainable part of policy systems, in other words, how it has been institutionalised. Kuchenmüller et al. [44] have synthesised this literature to describe domains of institutionalisation, and indicators of its progression. However, this conceptualisation pays less attention to how that progress is achieved. With this in mind, we conducted this retrospective ethnography to identify how evidence for policy has been institutionalised in the fields where we have direct experience.

### The case of the EPPI centre

The explosion of statistical meta-analyses included, thirty to forty years ago, the National Perinatal Epidemiology Unit (NPEU), University of Oxford, developing a database of controlled trials of perinatal care [15], compiling systematic reviews [14] and coordinating new trials [13]. This work led to the international Cochrane Collaboration, which extended systematic reviewing across health conditions [4]. Meanwhile, Ann Oakley adapted these methods to social interventions, first at NPEU, and subsequently at the Social Science Research Unit, Institute of Education, University of London.

This work began in 1993 with successive short-term contracts to create a database of evaluations of social interventions and to synthesise the findings of education or health research; some contracts were funded for their contribution to social science and some to inform policy decisions. Advances in methodology and information technology increasingly facilitated and safeguarded analyses of large literatures, and developments in collaborative working tailored analyses to meet the information needs of government rather than conform to traditional disciplinary boundaries in academia. Oakley et al. [67], in their early history of the EPPI Centre, focused predominantly on the resources for supporting evidence-informed policy, the technical and methodological challenges when building a cumulative evidence base, and the culture of academia and research funding. The future of this emerging field of research synthesis was uncertain, with questions remaining about the commitment of social science funders, social scientists and policy makers to invest effort and resources into working with evidence.

Nevertheless, the EPPI Centre continues to publish increasing numbers of systematic reviews, raise its profile in academic and policy circles both nationally and internationally, and expand its scope to encompass research across social science, into social care, global health systems, development studies, environmental science, crime and justice, and more.

As the global use of evidence for decisions has grown, we analyse here the increasing demand for evidence, how it has become embedded in policy development, and its impact on the wider world. Our study does not encompass all the EPPI Centre’s work. Rather we reflect on our own history at the EPPI Centre to ask what has supported and sustained our innovations for producing and using research for decisions, and with what impact.

## Methods

Having recently joined an international partnership that aimed to develop mechanisms and capacities to strengthen systems that support evidence-informed decision-making about social policy in the global south,^1^ we conducted this retrospective ethnography of our own experience to understand how evidence for policy has been institutionalised in the fields where we have direct experience.

### Theoretical framing

Two theories focusing on sustained change underpinned our work. Transition theory explains the initial stage, how changes emerge and are first taken up institutionally, while Transformative Social Innovation theory places more emphasis on how changes spread.

Transition theory [29] explains how sustained changes arise from technical innovations interacting with social factors at three different levels. Radical innovations emerge from niches (such as research centres) that interact with socio-technical regimes (such as universities, research councils or government departments) and their established practices and rules that stabilize existing systems. Both niches and regimes are influenced by wider sociotechnical landscapes. Transition theory posits that innovations developed within niches break through to influence regimes when the regimes are under pressure from cultural, economic or political changes in the wider landscape.

A second theory linking innovation and sustainable change is Transformative Social Innovation theory [111]. Based on insights from transition theory, social movement theory and institutional theory, empirical evidence of social innovation in multiple contexts, and direct practical experience, it is well suited to investigating the social movement of evidence for policy. Strasser et al. [112] portrayed transformative change in terms of the reach of new ideas (geographical and cultural spread), their societal depth (structural and cultural embeddedness) and sustainability (endurance and continuing evolution).

Merging these two largely overlapping theories guided our investigation of:

- niche activities (in this case the EPPI Centre) where innovations were incubated;
- organisational regimes (such as government departments, international organisations);
- interactions between the two, whereby new ideas were taken up and embedded in organisational regimes (breakthrough events); and
- the spread of innovations culturally and geographically as social movements.

The aim was to understand how we and our collaborators have contributed to the development of evidence and policy systems.

### Autoethnography

We conducted this analysis as three long-standing members of the EPPI Centre, making it insider research, where researchers have a direct involvement or connection with the research setting [95], especially over many years, when it may be considered ‘deep insider’ research [23]. Insider research benefits from having investigators who are familiar with and sensitive to the relevant contexts, cultures, histories and current debates.

We chose autoethnography [49] as an extension of our earlier reflexive insider research about working across the research-policy interface [71, 77]. Autoethnography draws on memories of events, hindsight for interpreting personal experiences, and contemporaneous documents and academic texts to illuminate organisational and wider cultures [1, 25]. Research rigour is enhanced by adopting a team approach for collecting and analysing data, with each researcher bringing different disciplinary backgrounds and organisational experiences, and challenging each other’s personal interpretations [49].

Sandy Oliver joined the EPPI Centre in 1995, bringing an academic background in virology and voluntary sector experience of evidence-informed activism. In 2004, Kelly Dickson, a sociologist, arrived from local government to support review teams embarking on reviewing education research and complemented her growing systematic review expertise with psychotherapy and an interest in working relationships. Joining a year later, Mukdarut Bangpan came with qualifications in business and experience of teaching adults, which encouraged a supportive rather than didactic approach to strengthening reviewing skills. We are only three of the 50 + members of the EPPI Centre who, since 1993, have brought a broad range of disciplines: sociology, social policy, music, philosophy, biology, nursing, geography, education and history of science. This autoethnography reflects our different experiences before and during our EPPI Centre work, each of us having worked with different combinations of EPPI Centre members over the timescale of this project. We would expect other colleagues to draw out other insights.

### Data collection and analysis

In adopting autoethnography, the personal (auto) element began with interviewing each other about our personal histories before and after joining the EPPI Centre, our working relationships with colleagues, collaborators and funders, and our cognitive and emotional responses to the need for and consequences of developing innovative research methods. Starting with our personal stories encouraged a reflexive approach to the paper, where we have taken account of how our partial viewpoints may influence what each of us can see, both positively and negatively, in our working context.

Our initial descriptive analysis (-graphy) emerged as we plotted key events in our individual and shared biographies on a paper timeline four metres long representing the years since 1990, when Oakley established the Social Science Research Unit at the Institute of Education, and began a stream of work systematically reviewing social science in 1993.

We sought a deeper understanding of our cultural (ethno) experience of working close to the research-policy interface by placing these events against a backdrop provided by the three levels of transition theory, noting interactions of the EPPI Centre (niche level) with key organisations and their wider influence at the regime level, and broader socio-political changes at the landscape level.

Transformative Social Innovation theory [111, 112] prompted us to recognise novel ideas emerging at the niche level that: deepened understanding, challenged established ways of working, or developed solutions with regime level institutions; spread across geographies, sectors, and population groups; and sustained change by embedding innovation and enhancing resilience, while evolving core characteristics and maturing.

Finally, with relevant micro and macro changes identified, we highlighted in our multi-level timeline the breakthrough moments recognised by transition theory when new developments incubated at the niche level were taken up by regime level institutions, and hypothesised the mechanisms of change.

Coloured marker pens distinguished the activities of niche and regime organisations, and landscape events. Coloured sticky notes indicated ideas developing, spreading and embedding. This visual representation allowed us to recognise breakthroughs as ideas developed at the niche level, subsequently influencing institutional regimes. For each novel idea breaking through, we sought mechanisms that enabled the breakthrough. We used this timeline to structure a narrative, checking our memories and drawing on earlier insights by consulting contemporaneous documents about our research and collaborations (usually not in the public domain, such as minutes of steering group meetings, end of grant reports and independent peer review by policy makers and academics), the impact of this work (documented by submissions to HEFCE for the Research Excellence Framework, 2014 and 2021), and publications by others about the wider context of two social movements, evidence for decision making and public involvement in research.

Insider research, autoethnography in particular, raises ethical challenges about how to protect participants and their associates and colleagues [21, 24, 49]. Therefore, working with the international PEERSS partnership we developed guidance for navigating ethical challenges that arise with collective efforts to advance understanding from professional and organisational experience. Embarking on an autoethnography of our professional lives may open us to personal criticism, not just intellectual critique of our outputs. Recognising that anonymity of the authors or organisation is not possible for a paper such as this, we adopted the common autoethnographic practice [25] of inviting EPPI Centre colleagues to read and comment on our work in progress. This work was approved by UCL’s Faculty of Education and Society Research Ethics Committee (REC 1621).

## Findings

**Fig. 1:**
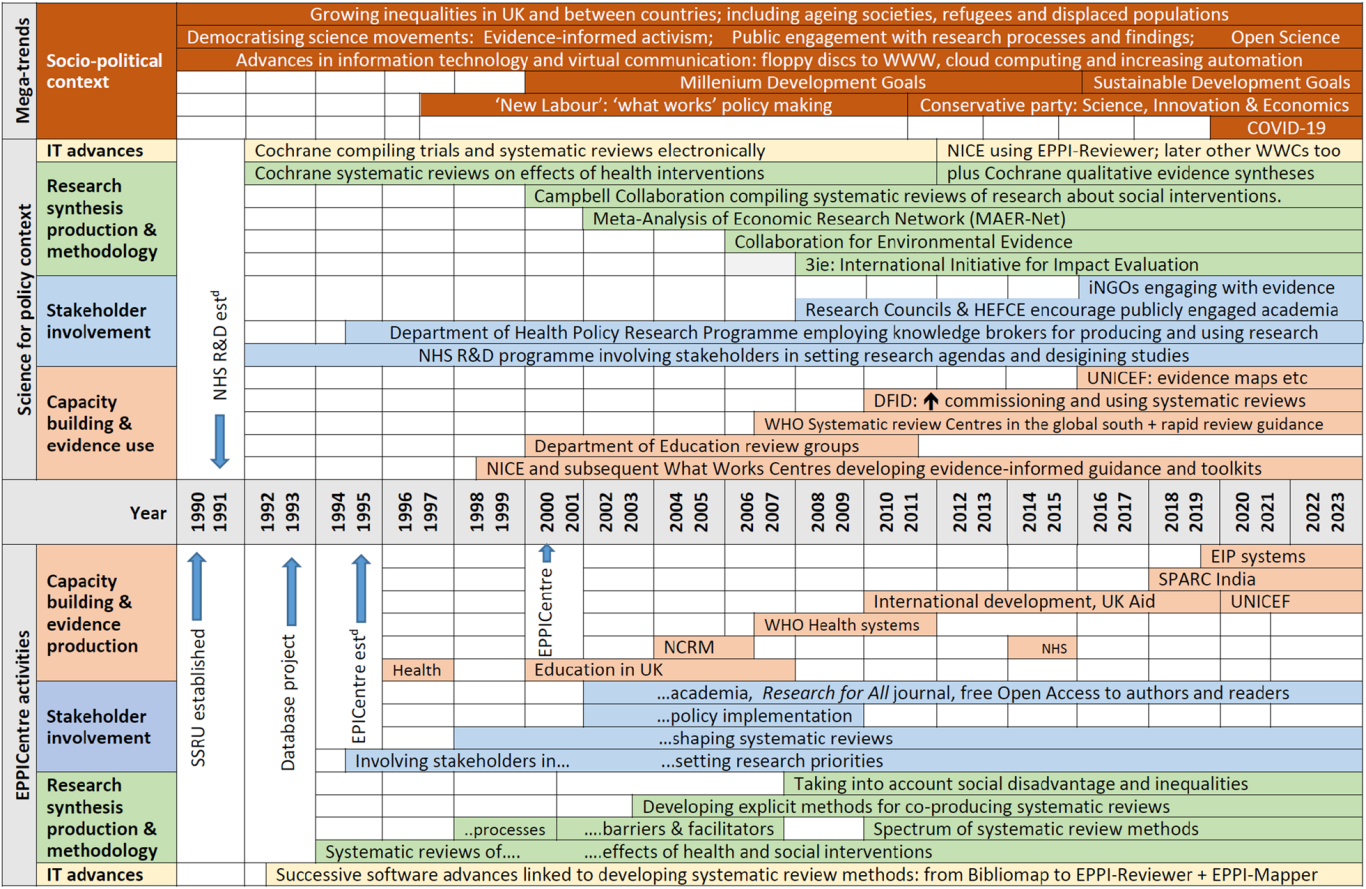
Timeline of EPPI Centre activities, their science and policy context, and global megatrends

Figure 1 presents EPPI Centre (niche level) activities, aligned chronologically with changes in the organisational sphere (regime level) of science and policy, and with global socio-political megatrends (landscape level). It shows the EPPI Centre at the forefront of innovations in both systematic review methodology, particularly in social science, and stakeholder involvement, a pioneer in ‘open science’, ahead of institutional incentives for external engagement, and working with national and international policy organisations for strengthening capacity and collaboration in systematic reviewing (activities that moved online during the pandemic, supported by university IT infrastructure and urgent work was unimpeded by the time and financial costs of international travel). In the following sections we explore how these innovative practices emerged, and how they were widely shared, taken up and sustained by academic and policy organisations.

### Incubating innovation

In 1990, when Oakley established the Social Science Research Unit at the Institute of Education, University of London, she was adapting methodological advances made in clinical science to study the processes and effects of social interventions. Primary evaluations and systematic reviews of social interventions faced similar methodological problems: the significance of randomisation and blinding, the importance of context, and mixing qualitative and quantitative methods [10, 62, 68, 76, 116].

By 1995, Oakley’s systematic review team had sufficient experience and on-going funding from the Department of Health to be recognised as the Centre for the Evaluation of Health Promotion and Social Interventions (EPI-Centre). It doubled in size in 2000, to become the EPPI Centre and adapt methods and strengthen capacity for the Department of Education. An academic base in London gave easy access to stakeholders with London offices for national public and voluntary sector organisations, and to bibliographic databases and journals in nearby libraries. The latter benefits were magnified when the EPPI Centre became part of a multidisciplinary university, University College London, in 2014, and as libraries offered on-line access. More difficult in the short term was competing for external funds with more nimble independent consultancies to provide rapid reviews to meet urgent policy deadlines.

Niche organisations typically participate in a small social network of committed entrepreneurs or innovators willing to take a chance, employed in precarious structures and investing considerable time and energy in upholding the niche [29] – just like the EPPI Centre, funded largely with short term contracts. Innovative practices developed in this way are typically restricted to niche level attention for a long time, possibly decades. This may be because development and troubleshooting takes a long time, or because there is a mismatch with, or even resistance from, the existing regime where infrastructure or the complementary user practices have yet to develop [29].

The EPPI Centre experience was very different. During its early years (1993–6) incremental changes adapted methods from medicine to social science. The Cochrane model of tightly specified questions about clinical effectiveness that were narrow enough for practitioners to address on a voluntary basis was only slightly amended to evaluate social interventions such as sexual health education or preventing older people falling. However, these incremental changes were often insufficient because reviewing the nascent trials literature in health promotion produced reviews of effectiveness with too little evidence for policy development [65, 66, 89].

Following these early disappointing findings, rather than abandon reviews of social science, the Department of Health asked broader questions that demanded greater innovation. They asked for more attention to be paid to policy or programme implementation [33], as did commissioners and practitioners of health promotion services [79, 80, 83]. They asked the EPPI Centre to address other types of questions. Rather than answering questions about what works with controlled trials of interventions, they asked questions about what influences behaviour. So we chose to tackle the challenge of reviewing studies of young people’s views and combine the findings from these studies with the findings of sound trials and preliminary outcome evaluations. Our user-centred approach involved negotiating each review question with policy teams wanting to use the findings. Within six years, we had published a series of ‘barriers and facilitators’ reviews that offered policy teams evidence about effective (and ineffective) programmes, and promising interventions that deserved further evaluation. We also adapted systematic methods to deliver social science reviews more rapidly [117, 123].

We were not alone in developing new ways of working, although the various niche organisations operating in this field took some time to understand each other’s approaches. Debating the challenges and options for synthesising social science (often qualitative studies) with other social scientists clarified the similarities and peculiarities of different approaches. Some synthesis methods (e.g. meta-ethnography, grounded theory, critical interpretive synthesis) produced more academic or theoretical findings, while other methods developed at the EPPI Centre (thematic synthesis, framework synthesis) better suited policy makers and designers of interventions [8]. Indeed, framework synthesis, as developed at the EPPI Centre, was designed with policy makers, to accommodate policy questions where theory was nascent, by involving various stakeholders in discussions to understand complex situations and frame the analysis [12, 78, 84]. We found that piloting new ways of working was essential for many systematic reviews addressing policy and practice priorities. Over time, these included developing acceptable interventions [79, 80, 83] and identifying their active components [116],and investigating inequalities in health [41] and reducing them through community engagement [88].

This collaborative approach [76] made research findings available before publication to policy teams who had commissioned them and others [9, 40, 70]. Within five years, this work also informed the Home Office research report on the drivers of perceptions of anti-social behaviour [54], and the Joint World Health Organization / International Labour Organization [126] policy guidelines on improving health worker access to prevention, treatment and care services for HIV and TB. In theoretical terms, this accelerated use of research was possible because regime level users and niche level producers collaborated to tailor innovations to meet user needs – uptake was anticipated the moment innovation was contemplated. The innovation incubator spanned the niche and regime organisations. Research and policy teams collaborating to ask atypical systematic review questions and answer them with novel methods was supported initially by knowledge brokers employed within the Department of Health’s Policy Research Programme. These knowledge brokers understood and respected the working practices and constraints of both research and policy teams. They encouraged research teams to draw out recommendations for policy, while also allowing them to abide by fundamental research principles.

All these reviews were supported by successive improvements in information technology. Initially on personal computers and floppy discs in the mid-1990s, the proximity of day-to-day reviewing and software development led to EPPI-Reviewer, which is particularly well suited to managing studies throughout the review process, including coding, analysis (whether statistical or thematic) [113], 2020) and text mining to accelerate the process. A more recent innovation is EPPI Mapper software^2^ for visualizing review results Digital Solution Foundry and EPPI-Centre [20].

Developments for working collaboratively and rapidly to synthesise social science literatures were particularly valuable for responding to urgent evidence needs prompted by the COVID-19 pandemic. Early reviews addressed immediate challenges: the impacts on child protection [6],and palliative care at home [27]. Other priorities soon arose, demanding evidence about: mental health issues arising during COVID-19 [19],equity and policies for schools [105],and mobilising volunteers [43]. Several of these reviews drew on two living evidence maps developed by colleagues during the early months of the pandemic: a map of COVID-19 studies [51, 103],and another of social science systematic reviews of COVID-19 [102].

New ways of working evolved from similar collaborative approaches for developing patient involvement in setting research agendas, starting with pilot studies conducted with patient advocates and the NHS Health Technology Assessment (HTA) programme [79, 80]. Broadening the scope to setting agendas for NHS research and development required new methods for systematically reviewing another emergent literature. These methods were developed by a team comprising the EPPI Centre as a niche organisation, the National Coordinating Centre for Health Technology Assessment as the regime organisation, and patient advocates [73, 78, 84]. The findings first informed the HTA programme, and subsequently a widely used guidebook for setting research priorities [17].

Key to our success, in both research synthesis and public involvement, was working collaboratively across the interface of research and policy.

### Spreading innovation

These new ways of commissioning and producing evidence for decisions soon spread across policy sectors, across academic disciplines and across geographies, following pathways recognised for spreading new ideas through service organisations more broadly [32]. The EPPI Centre’s approach of co-producing systematic reviews had the relative advantage of generating evidence that was policy-relevant and compatible with stakeholders’ values, norms, and perceived needs.

We initially shared ideas about social interventions evidence by adapting Critical Appraisal Skills Programme (CASP) workshops for health promotion with the help of prospective participants and their peers [82]. Feedback from one of these workshops re-shaped a Cochrane review [53, 79, 80, 83]. Similarly, participatory workshops for policy makers, practitioners and researchers from southern Africa in evidence-based decision making for HIV included feedback sessions to refine training materials for the following day [110]. When the Social Care Institute for Excellence (now part of NICE) shared examples of stakeholder involvement, they included our collaborative approach to shaping systematic reviews [92] and using them to develop policy [109].

Around the same time, the concept of informing professional practice with research entered teaching [34]. Hargreaves’ call for developing teaching as a research-based profession, and developing research to address stakeholders’ priorities, was soon followed by our first large scale capacity strengthening programme. Commissioned by the Department for Education between 2000 and 2010, the EPPI Centre provided methodological guidance and support to authors of more than 50 systematic reviews on a range of education topics. The tools we had developed for reviewing health promotion research were considered inappropriate by some education researchers until, following challenging discussions, we adapted both tool content and language [26]. Wider sharing of synthesis methods was through an EPPI Centre lead hub of the ESRC’s National Coordinating Centre for Research Methods (2005–2008)^3^ and, later, a book that introduced both the collaborative and technical aspects of systematic reviews [30].

The EPPI Centre grew a reputation in systematic review and policy networks for shaping questions and interpreting findings collaboratively with review users, which attracted the attention of organisations whose interests did not fit ‘the medical model’ that was widely perceived to be driving systematic review methodology. First the Alliance for Health Policy and Systems Research approached us about evidence on health systems rather than health practices, particularly in the global south. They encouraged nationally-driven, interpretive approaches to setting research priorities with a range of stakeholders [90]. Alongside other academics, we supported them as they commissioned centres in the global south to deliver portfolios of systematic reviews addressing those priorities [48]. They particularly valued rapid reviews, and collated the accrued learning in a handbook [118]. Subsequently, the UK Department for International Development (DFID), also with a global south remit, invited us to join their systematic review programme to inform socio-economic development more broadly. These were both capacity strengthening programmes ‘close to policy’, supporting research teams to develop reviewing skills in their academic areas of interest [72]. An external review of the DFID programme recommended improving systems for identifying questions, stakeholder engagement, knowledge brokerage, and policy team commitment to the review process [96]. The Alliance’s interest in addressing systems and DFID’s interest in addressing broad policy questions required the EPPI Centre to work with novice review authors to clarify and operationalise the early stages of systematic reviewing: setting the questions and developing conceptual frameworks to support the analysis. Our earlier work, where we adapted framework analysis [93] to systematic reviewing [78, 84], proved particularly helpful for supporting review and policy teams construct frameworks for reviewing emerging literatures more generally [71, 77]. Supporting dozens of review teams provided ample opportunity to encourage policy-relevant reviews, and the DFID-funded reviews subsequently influenced many policy decisions within DFID and other organisations [70].

The Alliance and DFID invested not in individual reviews, but in portfolios of work that required on-going methods development. The spread of this work was driven not by uptake of findings from specific systematic reviews, nor by uptake of specific research methodologies, but by uptake of the principle of learning from research literatures rather than individual studies alone. How to apply that principle in different substantive areas was unclear in advance. These funders were willing to apply systematic review methods to new fields with no guarantee of success. The Alliance established systematic review centres in the global south with the support of academics specialising in tropical medicine, health systems and social science [2]. DFID’s later programme of systematic reviews, with integrated capacity building, addressed their own priorities with the support of academics from education, health, social policy, development studies and environmental science.^4^ In each case, the EPPI Centre continued to develop its user-centred approach to collaboratively conducting systematic reviews and developing review methods when necessary.

In time, the interpersonal aspects of working with potential review users were refined and codified as a core aspect of the research method [71, 77]. What was institutionalised was setting review priorities with potential review users, either within government or outside with multiple stakeholders, with each review guided by potential users. This was not uptake of technological solutions but uptake of a collaborative research approach to innovate at the interface.

Early legitimacy for the EPPI Centre, in a field dominated by medicine where research synthesis was widely accepted, came from Oakley securing Medical Research Council funding for studying behavioural interventions [66] at a time when there were no effective treatments for HIV/AIDS. In contrast, taking research synthesis into new substantive areas and disciplines challenged existing ideas. We met strong opposition to randomised controlled trials and systematic reviews of social interventions, with objections expressed in terms of methodology, practical challenges and professional claims to expertise [63, 64, 67, 89, 115]. Transition theory acknowledges the resistance of institutions, networks and organizations who have a vested interest in maintaining the status quo [97]. Indeed, Hargreaves [34] predicted opposition to evidence-based education from academics who may fear an applied agenda would result in a loss of autonomy and control of the research process. Five years later, meeting such opposition was personally uncomfortable for the whole team. As early career researchers, for instance, adapting a methodology to the field of education prompted interdisciplinary disputes with leading professors. Systematic reviews, particularly those by the EPPI Centre, were seen as unscholarly [55], and incompatible with orthodox education research that was largely qualitative, phenomenological and operating at the grassroots [3]. We had already encountered opposition beyond academia, in 1996, when the Department of Health commissioned us to deliver workshops to encourage evidence-informed approaches. There was ‘a wide range of attitudes towards evaluation [amongst those funding or delivering] health promotion interventions and often a very political atmosphere with heated discussions; competition between the organisations attending was also apparent’.^5^ This competition was stoked by their commitment to reducing transmission of HIV/AIDS with a limited pool of funds for public and voluntary sector programmes.

Entering new substantive fields exposed gaps in our own professional backgrounds. We felt isolated by criticism coming simultaneously from colleagues who accused us of abandoning methodological principles, and from practitioners who considered us unrealistic and ill-informed. External hostility strengthened internal team bonds and encouraged a pioneer spirit (Oakley, personal communication). Ironically, opposition was typically provoked by capacity strengthening programmes, first with health promotion specialists in the 1990s, then with education academics starting in 2000, and from 2010, in the field of development studies. EPPI Centre commitment was tested when early career researchers made extensive contributions to raise the rigour of work authored by novice systematic reviewers, while also meeting strong criticism and losing opportunities to prepare publications that would support their own academic careers. It took persistence to maintain a constructive ethos that assumes everyone in a field has something to offer and everyone has a reason to listen.

Looking back, this ‘opposition’ may be better understood as tensions arising from various professions, activists and academic disciplines meeting to address shared interests while bringing their different knowledge, values and histories. When explicitly commissioned to encourage greater production or use of systematic reviews by specialists in health promotion, education, health systems and socio-economic development, our simultaneous challenge was to understand policy and practice priorities, and research practices in unfamiliar fields.

Over time, in the health field we were able to combine randomisation and qualitative research into a single review, to learn more about children and healthy eating from the two together than either separately [116]. In education, early opposition is now largely replaced by acceptance, with systematic reviews featuring in a chapter of *The BERA/SAGE Handbook of Educational Research* [87], a flurry of reviews about schools during the COVID-19 pandemic, and a call for systematic reviews to appear in a special feature of the *London Review of Education*.^6^ One breakthrough here was an ex-schoolteacher at the EPPI Centre, Janice Tripney, accruing hands-on experience of reviewing research about technical and vocational education and training for young people in low- and middle-income countries. Tripney collaborated with economists who were advancing novel methods in the field of international development that embraced a wider range of study designs: experimental designs and quasi experimental designs based on statistical methods. Their work appeared in journals specialising in systematic reviews [120] and education [121]. At the same time, others in the field of international development overcame practical and theoretical obstacles to tailor review methods to wider problems or questions, and their relevant literatures [45, 127]. All these are examples of new ideas developing when the EPPI Centre and others responded to policy organisations being ready to adopt the principles of systematic research synthesis. Rather than spreading new methods for policy-relevant reviews, it was exchanging ideas between research and policy that developed those methods in new spheres of work.

In summary, spreading innovations, in this history, is better described as sharing the innovation process with wider networks and regime organisations. Uptake of innovations is better described as taking up the innovating team, whose continuing innovations were inspired by working for and with the regime organisations. Incubating and spreading innovations have been inextricably linked.

### Embedding innovation

As systematic reviews grew in number and influence, specific approaches to reviewing health research became institutionalised as the international Cochrane Collaboration. However, the EPPI Centre’s mid-1990s focus on health promotion, such as healthy eating and physical activity, was a poor fit with Cochrane’s fast developing structures that differentiated health conditions, such as heart disease or musculoskeletal problems. Although the EPPI Centre made early contributions to the Cochrane Collaboration, through the Cochrane Field for Reviews of Behavioural Research and Health Promotion, this ‘field’ structure was excluded from hosting and editing reviews. Instead, EPPI Centre ways of working were assimilated by policy organisations. The first EPPI Centre project embedded in a policy organisation built on our expertise of working with multiple stakeholders; it produced systematic reviews and took their findings into account when collaboratively designing and implementing a national programme of newborn screening for rare but serious metabolic conditions [107]. The parent information and professional training resources were well received by clinicians, “well used and valued by both women and midwives” in the UK, and adopted by many programmes around the world [39].

Other collaborative partnerships produced systematic reviews ‘in house’ to inform policy or professional practice, or embedded EPPI-Reviewer to support their evidence work. Government social researchers co-authored a rapid evidence assessment tool kit for civil servants with the EPPI Centre.^7^ In 2006, the EPPI Centre participated in methodological discussions when NICE was expanding its remit to include public health [85, 86] and social care [18]. The NICE manual for developing guidelines [60] had EPPI Centre membership of the group that advised its development, and the current version cites five EPPI Centre methodological publications [60]. Similarly, when UNICEF developed methodological briefs about evidence synthesis products to strengthen decision-making within the organisation, it invited EPPI Centre input and cited several EPPI Centre papers to guide their work [5]. In time, despite the differences in framing health topics of interest, the EPPI Centre also influenced Cochrane’s review methods through its Handbook for Systematic Reviews of Interventions, first by co-authoring chapters [37], and now co-editing the handbook itself [38].

Close collaboration between the EPPI Centre and NICE tailored EPPI-Reviewer for the purpose of analysing studies when developing guidelines. EPPI-Reviewer [115], and EPPI-Mapper software^8^ for visualizing the literature, are now used by several other What Works Centres. On an international scale, EPPI-Reviewer is one of two ‘official’ Cochrane tools (with a particular focus on complex reviews) and the EPPI-Mapper software was developed initially for Campbell Evidence and Gap Maps, and is now publicly available.

Being based in academia, we built on our professional development workshops for spreading awareness and skills for working with evidence to develop postgraduate courses that met institutional expectations for accreditation, and thereby embedded systematic reviews into academic structures while sharing the ideas even wider through an international student body. Once accredited courses were established, being able to move staff between research and teaching helped balance the unpredictability of short-term research contracts. Another mechanism for embedding social science systematic reviews in academia was collaborating with library staff to develop a cross-disciplinary guide to systematic reviews [106]. This opportunity to sustain academia’s interest in policy-relevant systematic reviews was less available to other small reviewing organisations that were predominantly funded by research councils or philanthropic foundations, remained independent to deliver products or services for decision-makers (e.g. Kleijnen Systematic Reviews Ltd),^9^ or were hosted by third sector (e.g. [94] or commercial organisations (e.g. Bazian).^10^ Notable exceptions included academic centres focusing on health that were embedded in the NHS R&D programme, such as the Centre for Reviews and Dissemination at the University of York, and the Wessex Institute at the University of Southampton.

Complementing efforts to embed evidence into policy decision making, were efforts to embed wider society into research decision making. With our strong record of involving stakeholders, including policy makers, to shape our own research, we were able to secure Research Council funding to encourage an organisational culture where excellent public engagement with research is formalised and embedded. This collaborative endeavour of eight universities supported by the National Coordinating Centre for Public Engagement [22] led to institutional support for establishing an international open access journal about ways for universities and communities, services or industries to work together for research.^11^

As with the spread of innovations, institutionalisation of EPPI Centre innovations was less about uptake and embedding of innovations, but more about co-developing and tailoring innovations with regime organisations, including our own university.

## Discussion

This autoethnography found that novel ideas and methods were not developed by the EPPI Centre or other organisations individually, but by organisations collaborating across the research-policy interface, comparing different ways of working and adopting inclusive approaches to transform conflict into consensus. Systems change came not from uptake of innovations, but by uptake of teams who innovate and teams having a mutual commitment either side of the research-policy interface. Embedding innovation came not from incorporating innovations, but from research-policy collaborations co-developing and tailoring innovations within regime organisations.

Incubating, spreading and embedding innovation has been iterative, with activities for spreading innovation inspiring further innovation; and embedding innovation requiring further innovation. Institutionalising evidence for policy required change in both institutions generating evidence and institutions developing policy.

### Strengths and limitations of this study

Two theories informing our analysis channelled the focus of our reflections to interactions between the EPPI Centre and other organisations in the field of evidence for policy. They helped to formulate ideas about pathways for incubating, spreading and embedding policy ways of working that are open to research, and research ways of working that are open to policy priorities.

As retrospective autoethnography relies heavily on the authors’ memory, some issues may have been missed. We mitigated the risks of poor recall by referring to our peer reviewed publications that recorded either our methodological developments or our disappointing findings. We also made efforts to confirm events and interpretation with colleagues and contemporaneous records of meetings. The risk of overstating our own contributions was mitigated by corroborating evidence (whether favouring or criticising our work) published by collaborators and others (indicated in Table 1 by ***bold italic font***). Three co-investigators with good rapport, differing perspectives and a strong track record of challenging each other’s ideas and interpretations through the conventional practices of double coding in systematic reviewing were all assets. These assets align with recommendations that Tripathi et al. [119] drew from the wider literature for conducting retrospective collective autoethnography.

**Table 1:** Findings and supporting evidence

Insights and lessons	Supporting evidence
Incubating innovation	
Key to successful innovation in research synthesis methods for decision making was working constructively across the interface of research with the wider world	**Conventional methods** minimally adapted for new fields, in this case methods for reviewing the effects of clinical interventions applied largely unchanged to reviewing health promotion interventions, provided scant evidence to inform policy or practice decisions [67] **Novel synthesis methods** were inspired by discussions with policy teams, practitioners and service users with the aim of drawing learning from the evidence available to: better develop, implement and evaluate health promotion interventions [33, 76, 79, 80, 83, 104], identifying their active components [116], investigating inequalities in health [41], and reducing inequalities through community engagement [88] **Information technology** was ‘designed from the bottom up’ by software developers working closely with systematic reviewers, initially within the EPPI Centre, and then with other organisations needing software to support systematic reviewing [114]
Successful innovation in research synthesis methods required sufficient time and collaborative learning to transform exchanges from mutual criticism of different academic, policy or practice lenses to mutual understanding and ultimately integrating different sets of knowledge into coherent research syntheses	**Mutual criticism and heated discussions** often resulted from short term encounters in the form of single researcher-led workshops or occasional meetings with service providers commonly led to heated discussions about competing intellectual positions (Minutes of steering group meeting, March 1996). An exception was the fourth in a series of workshops where strong criticism of a systematic review was collated in a letter to the lead author who subsequently updated the review in light of the criticism [79, 80, 83] **Collaborative learning** came from working relationships between producers and users of systematic reviewers sustained over several years, leading to synthesis methods being adapted for new fields such as health promotion (Oliver and Peersman 2001), *health systems* [47] and socioeconomic development [75]
Application of novel collaborative research synthesis methods accelerated uptake of findings and methods	**Speedy uptake of evidence** appeared to result from collaborative working, with evidence informing decisions appearing within very few years. The UK Newborn Screening Programme Centre (established in 2002) published its *UK national standards, policies and handbook within three years* [40], at the same time as its underpinning research about communication with parents and development of information and training resources [35, 36, 110]. *Pre-publication findings from a Cochrane review* [52] *informed NICE guidelines on smoking cessation in pregnancy* [9]. The literature about time lags in translational research in health [59] suggests this is faster than other estimates of the time between publication and guidelines, as calculated by Grant et al. (2000) and HERG (2008), which were eight years and 13 years respectively. This rapid uptake has become a feature of the more recent common practice of panels commissioning reviews to develop evidence-informed guidelines. For instance, a systematic review about home based records [56] informed the WHO guideline it was commissioned for the same year [124] and *health policy in Afghanistan only a year later* [99] **Speedy uptake of synthesis methods**: Similarly, novel methods co-developed with policy teams for reviewing health promotion [76] were applied independently within five years to inform (a) the *Home Office about the drivers of perceptions of anti-social behaviour* [54], and (b) the *development of guidelines about health worker access to preventive health measures* [125]
Spreading innovation	
Spreading innovation, in this history, is better described as researchers sharing the innovation process with wider networks and regime organisations	**Disseminating novel methods** had limited success. Stakeholder involvement to shape systematic reviews [92] and develop policy [109] was showcased by the Social Care Institute for Excellence (now part of NICE, https://www.scie.org.uk/almost-there) with evidence of use limited to six and one citations respectively (Google Scholar) **Disseminating systematic review evidence**: The Department for International Development placed systematic review reports in the public domain. Nevertheless, few of them appear to have been used unless they were also developed collaboratively with potential review users [70] **Collaboratively developing training** offered cycles of refreshing innovations for new audiences. Critical appraisal skills training, originally designed for clinicians, were adapted collaboratively with and for: consumer health information organisations [58]; health promotion organisations [82],and policy makers, practitioners and researchers in southern Africa [110] **Supporting collaborative learning** helped researchers and policy makers work together to produce systematic reviews that informed policy decisions; informing decisions happened more often when the working relationship with policy teams was acknowledged in review reports [70] **Research contributions to informing public policy**, recognised by the Robert Boruch Prize from the *Campbell Collaboration in 2015* (https://www.campbellcollaboration.org/the-robert-boruch-award)
Uptake of innovations, in this history, is better described as uptake of innovating teams, whose continuing innovations in research synthesis or public involvement were inspired by working for and with the regime **organisations**	**Evidence for education** was *stimulated by the Department of Education commissioning the EPPI Centre to support groups producing systematic reviews* [3] **Evidence for health systems** *gained from the Alliance for Health Policy and Systems Research (now hosted by WHO) commissioning the EPPI Centre (2007–15) to work with them to establish and support systematic review centres in the global south* [48] **Evidence for socio-economic development** *gained from the UK Department for International Development commissioning systematic review centres (2010–2019) to strengthen capacity in systematic reviews for socio-economic development* [70, 72, 96] and to develop collaboratively with the EPPI Centre a tool for assessing the impact of systematic reviews on governments and NGOs [70] **Evidence for humanitarian aid**: When developing their research methods guidance for health emergency and disaster risk management, *the World Health Organization* [126] *invited EPPI Centre authors to deliver the chapter on using logic models *in research and evaluation of health emergency and disaster risk management interventions
Embedding innovation	
Embedding innovations was less about uptake and embedding of innovative packages, but more about co-developing and tailoring innovations with regime organisations, including our own university	**Shared research agendas** resulted from collaborations between research organisations and patient advocacy groups. Patient and public involvement developed by a team within the Health Technology Assessment programme [79, 80] was subsequently given an expanded remit across the National Institute of Health Research. The *James Lind Alliance, which first worked independently with patients and clinicians to develop research agendas* [17], was later integrated into the NIHR **Evidence systems and guidance** for *regime organisations integrated new ways of working developed with the EPPI Centre and other partners* [2, 5, 38, 42, 47, 60, 126]. *Collaborative learning was applied again with PEERSS partners, which led to capacity-strengthening reforms in government departments in both the Caribbean and Brazil* [7] **Research infrastructure**: Our host university supported our collaboration with the National Coordinating Centre for Public Engagement, to establish a new diamond open-access journal (free access for readers and contributors) about methods for public engagement with research (*Institute of Education 2014 REF environment statement; OpenScience*https://uclpress.scienceopen.com/collection/UCL_RFA). These achievements were recognised by the *UCL’s Institutional Leadership Award for Public Engagement* in 2019. The EPPI Centre has similarly contributed advances in review methodology, information technology and research use to collaborative research infrastructure nationally and internationally (*UCL Education Unit environment statement 2021*) *Successful implementation of new policies:* UK standards, policies and handbook for newborn bloodspot screening resulted from a centre co-led by a clinician, clinical scientist and social scientist commissioned by the Department of Health in 2002. A participative model of public and practitioner involvement in evidence-informed policy to create a collaborative network for the development of national newborn blood spot screening policy in the United Kingdom [107]. Parent information and professional training for newborn screening (evidence-informed and co-designed) was well received by clinicians, “well used and valued by both women and midwives” in the UK, and adopted by many programmes around the world [39, 107])

*Italic* font indicates corroborating evidence from collaborators and others

This paper is unusual in focusing attention on both institutionalising evidence in policy organisations, and institutionalising stakeholder engagement in academia. Further work is required to explore each pathway and mechanism for the evolving system in more detail, yet bringing them together reveals important interactions between growing government interest in formalising the use of research and institutional changes in UK higher education.

These findings characterise the EPPI Centre as a knowledge brokering organisation; an organisation that shares decisions with mixed groups of people; and an innovative organisation that shares and embeds its innovations through research and policy networks. Here we discuss our findings in light of theories about these activities.

### Sharing information and decisions

Throughout our history, we relied on information sharing on a macro-scale, through extensive policy and research networks, and on a micro-scale, between policy and research teams working together for individual systematic reviews.

The theoretical literature about information flows, and bottlenecks, through social networks [50] includes studies about generating and integrating innovations. This literature describes innovation arising from knowledge brokers who have ‘the ability to select and synthesise different viewpoints and approaches from different, unlinked groups’ (p5), either drawing on the different knowledge held by those groups separately, or bringing them together to discuss their different viewpoints. Working in several evidence networks simultaneously gave us a vantage point of heterogenous knowledge developing in different disciplines, policy sectors and geographies where we could exchange different ways of working. A similar vantage point came from supporting dozens of novice teams conducting systematic reviews for broad policy questions. These relationships also brought strong ties and creative thinking that develops during collaborative working. This allowed us to take the principles of systematic reviewing from clinical science to health promotion, education, health systems, environmental science and socio-economic development.

On a micro-scale this autoethnography raises issues relevant to small group decision making. Making decisions that are far reaching, in our case designing systematic reviews, is more effective when a small representative group has sufficient time to develop trust and share, discuss and integrate their different sets of knowledge, preferably with the support of a skilled facilitator [71, 77]. While not all systematic reviews have these advantages, long term collaborations for systematic review programmes probably benefited, not only from the time that allowed the development of trust, but also time for us to develop knowledge brokering skills for facilitating discussions across the research policy interface.

### Institutionalising evidence for policy at scale

The EPPI Centre’s history has coincided with systematic reviews being ‘institutionalised’, in terms of their growing legitimacy in academia and increasing influence in policy decisions. In this section, we draw insights from the EPPI Centre’s history by considering six domains of institutionalising evidence for policy-making: resources; governance; standards and routine processes; partnership, collective action and support; leadership and commitment; and culture [44]. Our autoethnography reveals EPPI Centre innovations in all these domains. It also reveals the importance of institutionalising policy relevance in research organisations.

The EPPI Centre’s collaborative approach exemplifies the domain of partnership, collective action and support. Its leadership in the fields of social research synthesis, public involvement and information technology attracted interest from leaders in policy and research organisations to create influential collaborations. Academics willing to collaborate with external stakeholders have been recognised elsewhere by analyses of applied research networks for influencing health services [98] and university impact strategies more broadly [91]. Aligned with the findings of Reed et al. [91], this autoethnography portrays a research centre acting as a boundary organisation with a ‘bottom up’ approach to impact through co-production with key stakeholders.

The ‘culture’ domain ‘refers to basic values, assumptions, artefacts and beliefs which are considered valid and are being disseminated and promoted as daily practices’ [44]. When taking systematic reviewing to new academic disciplines and policy sectors, we had to take account of the values and literatures there, sometimes encountering criticism, resistance and conflict, particularly about rigour, relevance and being (un)realistic. For instance, moving from medicine to health promotion found fewer RCTs and a greater emphasis on process evaluations. Subsequently moving into international development, broad questions from policy makers needed correspondingly broad conceptual frameworks to manage extensive literatures spanning policy sectors and academic disciplines. Recognising that systematic reviews can rest on different theoretical and ideological assumptions [30] was important for tailoring them to different institutions. Working in an academic culture encouraged us to consolidate methodological advances in journal articles and doctoral theses.

Developing new ways of working frequently challenged values and assumptions. Ann Oakley’s pioneering of gender analysis [61, 62] inspired methods development to analyse inequalities [78, 84]. Oliver’s critiques of maternity services research [74, 81] and involvement of patient advocates in guiding research [69] were at the forefront of democratising research. The EPPI Centre’s outward facing research pre-dated HEFCE’s (2008) investment in universities to develop a co-ordinated approach to recognising, rewarding and strengthening public engagement. Publishing review reports and searchable databases on the EPPI Centre website pre-dated the Open Science movement at the beginning of the twenty-first century.

Studies of institutionalising evidence for policy have typically focused on policy organisations institutionalising evidence. Stewart et al. [108] drew attention to the complementary requirement to institutionalise policy relevance in research organisations. Working in a research centre in a university, our ongoing employment relied heavily on external funding from research customers, which encouraged very outward facing research. Key to retaining a critical mass of skilful staff were successive two to five year grants for systematic reviews in health (from the Department of Health since 1995), and for strengthening systematic reviewing capacity in education (10 years), health systems (8 years) and socio-economic development (8 years). The research funding supported detailed synthesis work, while funding for capacity strengthening provided opportunities to engage with a broad range of literatures. They both stimulated methodological advances in social research synthesis. We were bound by academic structures and procedures of our university, and more widely by the Higher Education Funding Council for England (HEFCE). HEFCE rewards universities for the originality, significance and reach of their research and the impact of that research on wider society, as assessed by the Research Excellence Framework.^12^ In addressing important policy questions, our reviews typically displayed originality in their methods as well as their findings. A small group of skilled academics could afford to place more emphasis on justifying innovations compared with larger systematic review organisations that emphasised routinized procedures for quality control. Yet, while remaining methodologically nimble with successive reviews, the EPPI Centre contributed to embedding routine procedures in larger organisations. Financial rewards came from documenting for HEFCE’s Research Excellence Framework how strengthening capacity for addressing important policy questions also informed policy decisions, professional practice and changed lives.^13^,^14^ Individual rewards came from promotion criteria that placed increasing importance on external engagement [122]. Ironically, the career pathways for navigating these criteria create sharp boundaries between academic, teaching and research contracts that, given the value of contract research for understanding the interface between academia and policy organisations, work against the principle of research-informed teaching.

In conclusion, applying theories of institutionalisation to the EPPI Centre’s history reveals the external structures, internal characteristics and events that were influential. It reveals the value of continuing innovation that builds on but is not constrained by commonly agreed standards and procedures. Taking into account the cultural domain, it emphasises the effort required to accommodate but also challenge established values and assumptions in policy sectors, academic disciplines and also systematic review methodology.

This autoethnography also shares some features with studies about spreading and scaling up innovation and improvement. It shows that developing policy-relevant systematic reviews is a poor fit with implementation science, which is described by Greenhalgh and Papoutsi [31] (p. 1) as taking ‘a structured and phased approach to developing, replicating, and evaluating an intervention in multiple sites’. It appears closer to complexity science, which they judge as encouraging ‘a flexible and adaptive approach to change in a dynamic, self organising system’ (p. 1). Our experience endorses the approaches these authors encourage: developing adaptive capability to refine technologies and processes; attending to human relationships that together can solve emergent problems; and harnessing conflict productively. We chose a social science approach to studying innovation and working at scale, applying transition theory to consider ‘why [we acted] in the way [we did], especially the organisational and wider social forces that shape[d] and constrain[ed our] actions’ (Greenhalgh and Papoutsi [31], p. 1). We recommend this approach to others working with evidence who wish to understand how and why their local systems have developed.

## Conclusions

As academics, our contributions to the development of evidence and policy systems began with developing research synthesis methods to address the problems and decisions faced in the wider world. This was done collaboratively with decision makers, and required working relationships developed over time to foster mutual understanding and thereby amalgamate different sets of knowledge into coherent research syntheses. The result was rapid uptake of both synthesis findings and methods by policy organisations. Working collaboratively with wider networks and regime organisations inspired further innovations as training and support for research synthesis spread. Indeed, uptake of new ideas by regime organisations was often achieved by working with the innovating teams. Embedding innovations was achieved by innovating teams co-developing and tailoring innovations with regime organisations.

Analysing our experience at the EPPI Centre has also revealed how incubating, spreading and embedding innovation in evidence for policy have overlapped and involved organisations collaborating from both sides of the research-policy interface in all stages. Key mechanisms for our contributions to advancing research evidence for decision making were: a commitment to changing public services through research; contract research for focusing attention at the research-policy interface; a willingness to work in unfamiliar fields; inclusive ways of working to move from conflict to consensus through developing long term collaborative relationships; and incentives and opportunities for reflection and consolidating learning.

## Data Availability

The dataset generated and analysed during the current study (namely an electronic version of the more detailed timeline) are available from the corresponding author on reasonable request.

## Footnotes

1 https://peerss.org/.

2 EPPI-Mapper https://eppi.ioe.ac.uk/cms/Default.aspx?tabid=3790.

3 https://www.ncrm.ac.uk/about/phase1.php.

4 https://www.gov.uk/government/publications/systematic-reviews-in-international-development/systematic-reviews-in-international-development. accessed 22 October 202.

5 Minutes of steering group meeting for ‘Field Coordination in Health Promotion linked to the Cochrane Collaboration’, SSRU, Thursday 28 March 1996.

6 https://cdn.shopify.com/s/files/1/1684/4803/files/Systematic_reviews_in_educ_CfP_2023.pdf?v=1671118635.

7 https://webarchive.nationalarchives.gov.uk/ukgwa/20140402164155/http://www.civilservice.gov.uk/networks/gsr/resources-and-guidance/rapid-evidence-assessment.

8 EPPI-Mapper https://eppi.ioe.ac.uk/cms/Default.aspx?tabid=3790.

9 https://www.systematic-reviews.com/.

10 https://www.bazian.com/.

11 https://www.uclpress.co.uk/pages/research-for-all.

12 https://www.ref.ac.uk/.

13 https://impact.ref.ac.uk/casestudies/CaseStudy.aspx?Id=44325#.

14 https://results2021.ref.ac.uk/impact/1bf01efb-8db6-4fa6-a132-25aa220efdd6?page=1.
